# Lack of evidence for Zika virus transmission by *Culex* mosquitoes

**DOI:** 10.1038/emi.2017.85

**Published:** 2017-10-18

**Authors:** Christopher M Roundy, Sasha R Azar, Aaron C Brault, Gregory D Ebel, Anna-Bella Failloux, Ildefonso Fernandez-Salas, Uriel Kitron, Laura D Kramer, Ricardo Lourenço-de-Oliveira, Jorge E Osorio, Igor D Paploski, Gonzalo M Vazquez-Prokopec, Guilherme S Ribeiro, Scott A Ritchie, Laura B Tauro, Nikos Vasilakis, Scott C Weaver

**Affiliations:** 1 Institute for Human Infections and Immunity, Institute for Translational Research, and Department of Microbiology and Immunology, University of Texas Medical Branch, Galveston, TX, USA; 2 Division of Vector-Borne Infectious Disease, Centers for Disease Control and Prevention, Fort Collins, CO, USA; 3 Arthropod-Borne and Infectious Diseases Laboratory, Department of Microbiology, Immunology and Pathology, Colorado State University, Ft. Collins, CO, USA; 4 Institut Pasteur, Department of Virology, Arboviruses and Insect Vectors, Paris, France; 5 Centro Regional de Investigación en Salud Pública-INSP, Tapachula, Chiapas, Mexico; 6 Department of Environmental Sciences, Emory University, Atlanta, GA, USA; 7 Wadsworth Center, New York State Department of Health, Albany, NY, USA; 8 Laboratório de Mosquitos Transmissores de Hematozoários, Instituto Oswaldo Cruz, Rio de Janeiro, Brazil; 9 Department of Pathobiological Sciences, University of Wisconsin, Madison, WI, USA; 10 Instituto Gonçalo Moniz, Fundação Oswaldo Cruz, Salvador, Brazil; 11 Instituto de Saúde Coletiva, Universidade Federal da Bahia, Salvador, Brazil and Instituto Gonçalo Moniz, Fundação Oswaldo Cruz, Salvador, Brazil; 12 Australian Institute of Tropical Health and Medicine, James Cook University, Smithfield, QLD, Australia; 13 Institute for Human Infections and Department of Pathology, University of Texas Medical Branch, Galveston, TX, USA

**Dear Editor,**

Since Zika virus (ZIKV) emerged in the Americas, major research efforts have been focused on identifying the mosquito species responsible for transmission. While almost all published results support *Aedes aegypti* and potentially *Ae. albopictus* as urban vectors, a recent article^1^ suggests that *Culex quinquefasciatus* may serve as a ZIKV vector in Recife, Brazil, a region that has experienced a high incidence of infection. Accurately identifying the vector of a pathogen enables public health agencies to implement appropriate control strategies and inform citizens of proper prevention measures. Additionally, establishing the vector for an emerging pathogen paves the way for researchers to advance our understanding of virus–vector interactions and pursue novel methods of control. In contrast, erroneously incriminating a vector could lead to misdirected use of limited government funds, diversion of research efforts and misinforming the public through misdirected media and educational programs.

Traditional criteria for arthropod vector incrimination include: (i) demonstration of feeding or other effective contact with pathogen’s host; (ii) association in time and space of the vector and the pathogen-infected host; (iii) repeated demonstration of natural infection of the vector and (iv) experimental transmission of the pathogen by the vector.^2^

For ZIKV transmission in the Americas, criterion 3 has been met only for *Ae. aegypti*, with detection of naturally infected mosquitoes with titers compatible with transmission competence in Mexico (reviewed in ref. 3) and Brazil.^4^ *Ae. albopictus* has also been shown to be capable of laboratory transmission^5, 6, 7, 8, 9^ (see also references reviewed in refs 3, 10). Although no field infections have been reported for *Ae. albopictus* in the Americas, they were detected during a 2007 Gabon outbreak (reviewed in ref. 3). In locations where *Ae. aegypti* has been found infected at high rates in the Americas, testing of *Cx. quinquefasciatus*, typically the most common urban tropical mosquito, was unsuccessful aside from three pools collected in Recife, Brazil described by Guedes *et al.*^1^ However, the ZIKV RNA levels measured in these Recife pools, reflected in high Ct values (37.6–38.15) representing <10 infectious units in typical RT-qPCR assays, are incompatible with transmission-competent mosquitoes, which typically have viral titers several orders of magnitude higher^5, 6, 7, 8, 9, 11^ (see also references reviewed in refs 3, 10). Even naturally infected mosquitoes without viral dissemination to the salivary glands typically have higher titers^7^ (see also references in ref. 10) than reported by Guedes *et al.*^1^ Thus, the wild-caught Recife mosquito pools likely contained trace amounts of residual, viremic blood in their guts, undetectible by their colorimetric assay, legs or other dislodged appendages from other infected mosquitoes of different species, or were false-positives.

In laboratory studies of *Cx. quinquefasciatus* other than those of Guedes *et al.*,^1^ only one other group has shown transmission of ZIKV by colonized mosquitoes, from an unreported generation number maintained since 2014.^12^ At least 15 other studies have found no transmission competence,^6, 7, 13, 14^ (see also references reviewed in ref. 3, 10) even after examining several combinations of geographic strains of mosquito as well as ZIKV, along with different methods of oral exposure. These include other studies from Brazil with colonies established in 2016 and another study from China.^7^ Even after intrathoracic inoculation, generally the most permissive route for arbovirus infection of mosquitoes, *Culex* mosquitoes were found to be refractory to disseminated infection (reviewed in refs 3, 10). Although some of these studies found infection of the midgut without dissemination to the saliva, the majority found no indication of any infection after oral exposure. Many of these ZIKV-refractory populations tested are highly competent for West Nile and St. Louis encephalitis flaviviruses, so the specific ZIKV block in these populations would need to be restricted to most but not all *Cx. quinquefasciatus* populations to explain the results of Guedes *et al.*^1^ In addition, *Cx. quinquefasciatus* from Recife challenged in another study with the same BRPE243/2015 ZIKV strain used by Guedes *et al.*,^1^ as well as with two other ZIKV strains, were consistently refractory to oral infection.^15^

The discrepancy between the negative results from so many published studies and the questionable findings of Guedes *et al.*^1^ should engender caution in interpretation and conclusions reported by media and public health authorities unless they are verified by more robust results including detection of genuinely transmission-competent mosquitoes in nature. Until further data are collected and other groups can replicate the Recife findings, it is important that public education and interventions remain focused on the conclusion supported by the vast majority of studies: *Ae. aegypti* is the only mosquito species for which we have strong evidence of ZIKV transmission in the Americas.

This submission represents the views solely of the authors and does not constitute those of the Centers for Disease Control and Prevention or the United States Government.
